# Insecticide susceptibility of *Aedes aegypti *and *Aedes albopictus *in Central Africa

**DOI:** 10.1186/1756-3305-4-79

**Published:** 2011-05-15

**Authors:** Basile Kamgang, Sébastien Marcombe, Fabrice Chandre, Elysée Nchoutpouen, Philippe Nwane, Josiane Etang, Vincent Corbel, Christophe Paupy

**Affiliations:** 1 Institut de Recherche pour le Développement (IRD), UMR 224 MIVEGEC, BP 64501, 34394 Montpellier, France; 2 Laboratoire de Recherche sur le Paludisme, Organisation de Coordination pour la lutte contre les Endémies en Afrique Centrale (OCEAC), BP 288, Yaoundé, Cameroun; 3 Centre de Recherche Entomologique de Cotonou (CREC), BP 2604 Cotonou, République du Bénin; 4 Centre International de Recherches Médicales de Franceville (CIRMF), BP 769, Franceville, Gabon

## Abstract

**Background:**

*Aedes aegypti *(Linnaeus, 1762) and *Aedes albopictus *(Skuse, 1894) are the main vectors of dengue (DENV) and chikungunya (CHIKV) viruses worldwide. As there is still no vaccine or specific treatment for DENV and CHIKV, vector control remains the cornerstone of prevention and outbreak control. Unfortunately, vector control programs are facing operational challenges with mosquitoes becoming resistant to commonly used insecticides in several areas through the world. Throughout Central Africa no recent data are available susceptible/resistant status of either vector species since the introduction/arrival of *Ae. albopictus *in this area. We therefore studied the level of resistance of these two major vectors to insecticides commonly used in Africa for mosquito control.

**Results:**

*Aedes aegypti *and *Ae. albopictus *were sampled in six urban localities of Cameroon (Garoua, Bertoua, Yaoundé, Bafia, Buea) and Gabon (Libreville). Larval bioassays, carried out to determine the lethal concentrations (LC_50 _and LC_95_) and resistance ratios (RR_50 _and RR_95_) suggested that both vector species were susceptible to *Bti *(*Bacillus thuringiensis var israeliensis*) and temephos. Bioassays were also performed on adults using WHO diagnostic test kits to assess phenotypic resistance to deltamethrin, DDT, fenitrothion and propoxur. These experiments showed that one population of *Ae. aegypti *(Libreville) and two populations of *Ae. albopictus *(Buea and Yaoundé) were resistant to DDT (mortality 36% to 71%). Resistance to deltamethrin was also suspected in *Ae. albopictus *from Yaoundé (83% mortality). All other field mosquito populations were susceptible to deltamethrin, DDT, fenitrothion and propoxur. No increase in the knockdown times (Kdt_50 _and Kdt_95_) was noted in the Yaoundé resistant population compared to other *Ae. albopictus *populations, suggesting the possible involvement of metabolic resistance to deltamethrin and DDT.

**Conclusion:**

In view of the recent increase in dengue and chikungunya outbreaks in Central Africa, these unique comparative data on the insecticide susceptibility of *Ae. aegypti *and *Ae. albopictus *could help public health services to design more effective vector control measures.

## Background

Dengue virus (DENV, *Flaviviridae, Flavivirus) *and chikungunya virus (CHIKV, *Togaviridae, Alphavirus*) are mosquito-borne viruses of medical concern in most tropical regions. With about 50-100 million reported cases annually, including 500 000 severe cases of dengue haemorrhagic fever (DHF) or dengue shock syndrome (DSS), DENV is the most prevalent mosquito-borne human virus worldwide [1]. In West and Central Africa, where DENV epidemics remained limited until 5 years ago, the number of outbreaks or case reports has increased significantly in several areas, including Cameroon in 2006 [2,3], Gabon in 2007 [4], and West African countries such as Mali in 2008, and Cape Verde and Senegal in 2009 [5]. Similarly, CHIKV, which previously caused only sporadic outbreaks in sub-Saharan Africa [6], has recently emerged in several urban epidemic foci in Central Africa [4].

*Aedes aegypti *(Linnaeus, 1762) and *Aedes albopictus *(Skuse, 1894) are the main epidemic vectors of DENV and CHIKV worldwide [2,7]. Both species occur in sub-Saharan Africa: *Ae. aegypti *is native to Africa and *Ae. albopictus *has recently invaded several Central African countries [8]. Recent observations in Cameroon [9] and Gabon [10] indicate that these two species infest urban environments.

As there is still no vaccine or specific treatment for DENV or CHIKV, vector control remains the cornerstone of prevention and outbreak control. Conventional control strategies rely on the reduction of larval sources by eradicating water-holding containers that serve as larval habitats, and by using larvicides (e.g. temephos and *Bacillus thuringiensis var israeliensis *[*Bti*]) in natural and/or domestic breeding sites [11]. Space spraying (using pyrethroids or organophosphates) is generally used when larval source reduction fails to reduce the density of adult mosquitoes, and also during outbreak situations [12]. Unfortunately, many vector control programs are threatened by the development of insecticide resistance in *Ae. aegypti *and *Ae. albopictus *[13-16]. Resistance to multiple insecticides (e.g. pyrethroids and organophosphates) has been reported in *Ae. aegypti *in South-East Asia [17,18], South America and the Caribbean [19-21]. There have been fewer reports of insecticide resistance in *Ae. albopictus *[15,22], although a significant decrease in susceptibility to permethrin, malathion and temephos has been observed in Thailand [14]. In West and Central Africa, most available data on pesticide resistance concern *Ae. aegypti *but date back more than 30-40 years [23], while virtually no such data are available for *Ae. albopictus*, owing to its recent introduction in Africa. In order to implement effective and sustainable arbovirus vector control measures, there is an urgent need to determine the susceptibility of the two major vectors of DENV and CHIK to insecticides commonly used for mosquito control. We therefore examined the distribution and insecticide susceptibility of both larval and adult *Ae. aegypti *and *Ae. albopictus *populations in sentinel sites from Central Africa (Cameroon and Gabon) using WHOPES-approved procedures [24,25].

## Materials and methods

### Mosquito sampling

**Figure 1:**
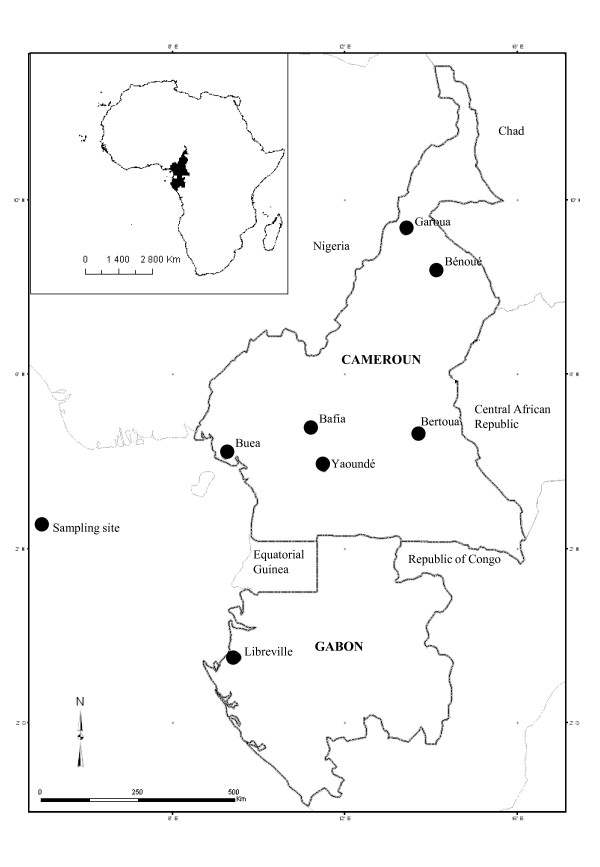
***Aedes aegypti *and *Ae. albopictus *sampling sites in Cameroon and Gabon**.

*Aedes aegypti *and *Ae. albopictus *mosquitoes were sampled as larvae or pupae in six urban localities (Figure 1): Garoua (09°16'N; 13°25'E), Bertoua (04°33'N; 13°46'E), Yaoundé (03°54'N;12°29'E), Bafia (04°44'N; 11°11'E) and Buea (04°09'N; 09°13'E°) in Cameroon in April 2007, and Libreville, Gabon (00°23'N; 09°27'E) in June 2007. An additional *Ae. aegypti *population (Bénoué) was sampled in the Bénoué National Park (08°15'N; 13°49'E). The latter population, which is sylvan and thus considered as naïve to pesticide, was used as a susceptible reference strain. Immature stages (field generation, F0) were collected from water in domestic (e.g. jars, tanks), peri-domestic (e.g. tires) and natural environments (e.g. tree holes). For each sampling site, larvae or pupae from 2-6 larval breeding places were collected, stored in plastic boxes and transferred to insectaries for rearing to the adult stage. Once identified as *Ae. aegypti *or *Ae. albopictus*, mosquitoes of the same species and from the same locality were pooled to prevent inbreeding. Mosquitoes were reared to generation F1 for larval and adult bioassays. Mosquito populations were maintained at insectaries conditions (27°C +/- 2°C; relative humidity 80% +/-10%) and females were fed on rabbits to complete their gonotrophic cycle.

### Larval bioassay

The susceptibility of larvae to temephos (organophosphate, Sigma Aldrich-Pestanal, Seelze, Germany) and *Bti *(VectoBac, Bayer CropScience, Monheim, Germany) was assessed using standard WHO bioassays [24]. Stock solutions and serial dilutions were prepared in 95% ethanol (temephos) or mineral water (*Bti*) and were stored at +4°C. Each bioassay used 25 late-third or early-fourth instars in plastic cups with 99 ml of mineral water and 1 ml insecticide solution at the required concentration. We used 5 concentrations in the range of activity of each insecticide and 50 to 100 larvae per concentration (with two to four replicates, depending on the sample and the number of larvae available). Each bioassay included a control group which received 1 mL of ethanol (for temephos) or 1 mL of mineral water (for *Bti*) only. Tests were run at 27°C ± 2°C, and mortality was assessed after 24 h of insecticide exposure. For each mosquito strain, one replicate corresponded to a single rearing batch.

### Adult bioassay

The procedure used for adult bioassays followed the standard WHO protocol [25]. Papers were impregnated using acetone solutions of insecticide and silicone oil as the carrier at the "Laboratoire de Lutte contre les Insectes Nuisibles" (LIN), Montpellier, France (WHO Collaborating Centre) using diagnostic concentrations defined for *Aedes *laboratory strain (Bora) susceptible reference strain [17]. Four technical-grade compounds representing the 4 major classes of insecticide (carbamate, organochlorine, pyrethroid and organophosphate) were used as follows: 0.3% propoxur (Bayer CropScience, Monheim, Germany), 4% DDT (Hindustan Insecticides Limited, New Delhi, India), 0.06% deltamethrin (Agrevo Environmental Health, Berkhamsted UK) and 0.5% fenitrothion (SUPELCO, Bellefonte, USA). Two to four batches of 25 non-blood-fed females (2-4 days of age) were introduced into exposure tubes containing impregnated filter papers for 60 minutes. The number of knocked-down (Kd) mosquitoes was recorded every 5 minutes. The mosquitoes were then transferred to a recovery tube containing 10% glucose solution and maintained at 27°C ± 2°C with 80% ± 10% relative humidity. Mortality was recorded 24 hours post-exposure. For each strain a batch of 25 mosquitoes was used as control.

### Data analysis

Larval and adult mortality rates were corrected using Abbott's formula (Abbott 1925) in case of control mortality >5% but less than 20%. Data from larval bioassays were analyzed with Win DL 2.0 software [26], which uses the iterative method of maximum likelihood to fit a linear regression between the log of the insecticide concentration and the probit of mortality. Goodness-of-fit was estimated with the Pearson χ^2 ^test. Win DL 2.0 provided also the slope of the regression lines and the estimates of lethal concentrations (LC_50 _and LC_95_) with their 95% confidence intervals (CI). Resistance ratios (RR_50 _and RR_95_) of each population compared to the Bénoué strain of *Ae. aegypti *(used as reference) were calculated as follows: RR_50 _= LC_50 _assay/LC_50 _reference; RR_95 _= LC_95 _assay/LC_95 _reference. A mosquito population was considered susceptible when RR_50 _was less than 2, potentially resistant when RR_50 _was between 2 and 5, and resistant when RR_50 _was over 5. For adult bioassays, resistant/susceptible status was defined according to WHO criteria [27]. Mosquitoes were considered susceptible if the mortality rates were greater than 97% and resistant if mortality rates were less than 80%. Mortality rates between 80-97% suggested possible resistance.

For deltamethrin and DDT, the knockdown times (KdT50 and KdT95) and their 95% confidence intervals were estimated with Win DL 2.0 software.

## Results

### Larval bioassay

Larval bioassays using *Bti *showed that LC_50 _and LC_95 _for *Ae. albopictus *ranged from 0.18 mg/l for the Bénoué strain (susceptible reference strain) to 0.69 mg/L for the Yaoundé and Buea populations. Against *Ae. aegypti*, *Bti *acted in a similar range of concentrations, with LC_50 _and LC_95 _ranging from 0.18 mg/L for Garoua to 0.91 mg/L for Bafia (Table 1). RR_50 _ranged from 1.00 (Garoua) to 1.50 (Bertoua) and RR_95 _from 1.13 (Libreville) to 2.84 (Bafia). These values and their 95%CIs indicated that all *Ae. aeggyti *and *Ae. albopictus *populations were similarly susceptible to *Bti*.

**Table 1:** Susceptibility of *Ae. aegypti *and *Ae. albopictus *larvae to *Bti*

Sample	N	Regression line	Pearson χ^2 ^goodness of fit	LC_50_ (95% CI)	LC_95_ (95% CI)	RR_50_	RR_95_
***Ae. aegypti***							
Garoua	493	Y = 5.67X + 4.15	0.69	0.18 (0.03-0.45)	0.36 (0.30-0.45)	1.00	1.13
Bertoua	498	Y = 3.84X + 2.13	0.62	0.27 (0.19-0.39)	0.75 (0.20-2.77)	1.50	2.34
Yaoundé	252	Y = 8.77X + 5.76	0.27	0.22 (0.20-0.44)	0.34 (0.26-0.44)	1.22	1.06
Bafia	500	Y = 2.99X + 1.75	0.54	0.25 (0.21-0.31)	0.91 (0.17-4.89)	1.39	2.84
Libreville	500	Y = 6.06X + 4.28	0.21	0.19 (0.09-0.24)	0.36 (0.32-0.46)	1.06	1.13
							
***Ae. albopictus***							
Yaoundé	500	Y = 1.91X - 0.04	0.64	0.19 (0.17-0.21)	0.69 (0.26-1.85)	1.05	1.06
Bertoua	477	Y = 16.31X + 9.32	0.94	0.27 (0.25-0.28)	0.34 (0.32-0.40)	1.50	1.13
Buea	375	Y = 3.08X + 2.13	0.51	0.20 (0.10-0.39)	0.69 (0.26-1.85)	1.11	1.06

***Reference strain***	496	Y = 7.04X + 5.11	0.40	0.18 (0.11-0.22)	0.32 (0.29-0.36)		

N: Total number of mosquitoes assayed; 50% and 95% lethal concentrations, LC_50 _and LC_95_, are expressed in mg/liter; 95%CI: 95% confidence interval; p > 0.05 suggests a well-fitting model, P < 0.05 suggests an invalid model population.

Against *Ae. aegypti*, bioassays showed that temephos LC_50 _values ranged from 0.004 mg/L (Garoua) to 0.009 mg/l (Bertoua) and the LC_95 _from 0.006 mg/L (Garoua) to 0.026 mg/L (Bertoua) (Table 2). RR_50 _ranged from 0.75 (Garoua) to 1.75 (Bertoua) and RR_95 _from 0.66 (Garoua) to 2.68 (Bertoua). These findings suggested that all the mosquito samples tested were susceptible to temephos. Concerning the Bertoua sample, although the RR95 value was over 2 indicating a suspected resistance, the IC values did not confirm this trend. LC_50 _values for *Ae. albopictus *ranged from 0.005 (Bertoua) to 0.008 mg/L (Libreville) and LC_95 _values from 0.008 (Yaoundé) to 0.017 mg/L (Libreville). RR_50 _ranged from 0.92 (Bertoua) to 1.43 mg/L (Libreville) and RR_95 _from 0.82 (Yaoundé) to 1.75 mg/L (Libreville), indicating susceptibility of all the mosquito populations to temephos.

**Table 2:** Susceptibility of *Ae. aegypti *and *Ae. albopictus *larvae to Temephos

Sample	N	Regression line	Pearson χ^2 ^goodness of fit	LC_50_ (95%CI)	LC_95_ (95%CI)	RR_50_	RR_95_
***Ae. aegypti***							
Garoua	500	Y = 10.68X + 25.82	0.36	0.0040 (0.0037-0.0042)	0.0064 (0.0060-0.0070)	0.75	0.66
Bertoua	500	Y = 3.57X + 7.28	0.48	0.0090 (0.0048-0.0170)	0.0260 (0.0022-0.3100)	1.75	2.68
Yaoundé	253	Y = 11.36X + 25.68	0.30	0.0055 (0.0052-0.006)	0.0076 (0.0067-0.0100)	1.04	0.78
Bafia	500	Y = 7.07X + 16.04	**0.006**	0.0053 (0.0047-0.0084)	0.0092 (0.0068-0.0410)	1.02	0.95
Libreville	500	Y = 8.17X + 19.56	0.061	0.0043 (0.0020-0.0081)	0.0068 (0.0051-0.0080)	0.81	0.70
							
***Ae. albopictus***							
Bertoua	493	Y = 6.75X + 15.58	0.14	0.0049 (0.0013-0.0180)	0.0086 (0.0061-0.0120)	0.92	0.89
Yaoundé	497	Y = 8.00X + 18.38	0.30	0.0050 (0.0021-0.0110)	0.0080 (0.0064-0.0100)	0.94	0.82
Buea	375	Y = 9.57X + 20.55	0.27	0.0071 (0.0065-0.0200)	0.012 (0.0087-0.0500)	1.34	1.03
Libreville	275	Y = 4.56X + 25.68	0.11	0.0076 (0.0023-0.0240)	0.017 (0.0017-0.1700)	1.43	1.75

***Reference strain***	500	Y = 6.22X + 14.17	0.57	0.0053 (0.0035-0.0079)	0.0097 (0.0073-0.012)		

N: Total number of mosquitoes assayed; 50% and 95% lethal concentrations, LC_50 _and LC_95_, are expressed in mg/liter; 95%CI: 95% confidence interval; p > 0.05 suggests a well-fitting model, P < 0.05 suggests an invalid model population.

### Adult bioassay

After 24 h post-exposure, one population of *Ae. aegypti *(Libreville) and two populations of *Ae. albopictus *(Buea and Yaoundé) were resistant to DDT (mortality 36% to 71%). DDT mortality rates suggested probable resistance in the Garoua *Ae. aegypti *population (97%) and the Bertoua *Ae. albopictus *population (80%). Probable resistance to deltamethrin was also detected in the Yaoundé *Ae. albopictus *population (80%). All *Ae. aegypti *except Yaoundé, and all *Ae. albopictus *populations were susceptible to deltamethrin, propoxur and fenitrothion (99-100%) (Table 3).

**Table 3:** *Aedes aegypti *and *Ae. albopictus *mortality rates 24 h after exposure to insecticides at diagnostic doses

	Mortality rate (%)							
	
	0.06% Deltamethrin		4% DDT		0.3% Propoxur		0.5% Fenitrothion	
				
	Assay	Control	Assay	Control	Assay	Control	Assay	Control
***Ae. aegypti***								
Garoua	100 (89)	2 (50)	96.8 (95)	8 (50)	100 (100)	8 (50)	100 (99)	2 (50)
Bertoua	100 (100)	2 (50)	-		-		100 (98)	2 (50)
Yaoundé	100 (95)	4 (50)	-		-		100 (96)	4 (50)
Bafia	100 (97)	4 (50)	-		-		100 (97)	4 (50)
Libreville	100 (97)	0 (50)	70.7 (99)	0 (50)	99 (100)	0 (50)	100 (99)	0 (50)
								
***Ae. albopictus***								
Bertoua	100 (80)	0 (50)	80.5 (87)	8 (50)	100 (94)	8 (50)	100 (61)	0 (50)
Yaoundé	83.3 (96)	2 (50)	36.3 (91)	12 (50)	100 (96)	12 (50)	100 (98)	2 (50)
Buea	100 (50)	4 (50)	47.0 (85)	2 (50)	100 (92)	2 (50)	100 (50)	4 (50)

***Reference strain***	100 (96)	2 (50)	-		-		100 (100)	2 (50)

Numbers in brackets indicate the number of mosquitoes assayed; -: sample not assayed.

The estimated knockdown times (KdT) for *Ae. aegypti *mosquitoes exposed to deltamethrin indicated that the KdT_50 _and KdT_95 _values of the field populations were similar (overlapping 95%CIs) to those of the reference strain (Table 4). KdT_50 _values recorded in Yaoundé and Buea *Ae. albopictus *strains were higher than for the reference strain. Despite the ICs calculated for these two strains did not overlapped with the IC of the reference, the increase in KdT seemed no significant (P > or = 0.05). The DDT KdT_50 _for all *Ae. albopictus *samples and the KdT_95 _for *Ae. albopictus *Bertoua were significantly higher than the corresponding values for all *Ae. aegypti *samples. These differences in KdT between *Ae. aegypti *and *Ae. albopictus *may be physiological and/or species-dependent.

**Table 4:** Knockdown times of *Ae. aegypti *and *Ae. albopictus *exposed to 0.06% deltamethrin and 4% DDT

**Locality**	***Ae. aegypti***		***Ae. albopictus***	
		
	**KdT_50_** (95%CI); P*	**KdT_95_** (95%CI); P*	**KdT_50_** (95%CI); P*	**KdT_95_** (95%CI); P*
		
	**Deltamethrin**		**Deltamethrin**	
Garoua	5.3 (4.7-5.9); P = 0.47	11.4 (9.7-14.2); P = 0.24	-	-
Bertoua	5.8 (3.3-8.2); P = 0.46	24.6 (16.6-51.4); P = 0.025	7.8 (7.3-8.3); P = 0.25	15.9 (14.4-18.0); P = 0.38
Yaoundé	6.7 (3.7-9.7); P = 0.35	22.8 (15.0-58.4); P = 0.05	11.2 (10.6-11.8); P = 0.06	17.3 (16.3-19.0); P = 0.28
Bafia	5.4 (3.3-7.4), P = 0.48	14.6 (10.1-36.9); P = 0.49	-	-
Buea	-	-	11.7 (10.2-13.3); P = 0.06	21.2 (18.1-27.4); P = 0.09
Libreville	5.9 (5.1-6.8); P = 0.45	13.4 (11.2-17.9); P = 0.40	-	-
***Reference strain***	5.5 (4.6-6.3)	14.5 (11.9-19.4)	-	-
				
	**DDT**		**DDT**	
Garoua	53.6 (49.9-59.2)	101.9 (84.0-146.0)	-	-
Bertoua	-	-	84.8 (72.8-106.9)	290.8 (200.7-523.0)
Yaoundé	-	-	77.4 (68.5-113.2)	109.2 (86.1-235.3)
Buea	-	-	68.0 (63.6-78.2)	93.9 (80.7-131.9)
Libreville	58.7 (55.8-62.7)	107.7 (94.6-129.2)	-	-

KdT: Knockdown times expressed in minutes; 95%CI: 95% confidence interval. *: P is the probability of homogeneity between the sample and the reference (Mantel-Haenszel chi square).

## Discussion

All *Ae. aegypti *and *Ae. albopictus *samples collected in Cameroon and Libreville (Gabon) were susceptible to *Bti *and temephos. Resistance to *Bti *has been described in *Culex pipiens *in the USA [28] and suspected in *Aedes rusticus *in France [29], but no resistance to this pesticide has been reported to date in *Ae. aegypti *or *Ae. albopictus *worldwide. Temephos is the most widely used larvicide for dengue vector control. Resistance to this organophosphate has been recorded in *Ae. aegypti *in Asia [30-34] and South America [35-37], and in *Ae. albopictus *in Malaysia [38] and Thailand [14]. Mouchet et al. [23] observed full susceptibility of *Ae. aegypti *larvae to temephos in Cameroon and Gabon (Libreville and Yaoundé) during the 1970s. Up to now, larvicides targeting these two species have rarely been used in African countries, thus explaining the persistent susceptibility to temephos and *Bti *in this part of the world.

WHO bioassays carried out on adult mosquitoes showed DDT resistance in one *Ae. aegypti *population from Gabon (Libreville) and two *Ae. albopictus *populations from Cameroun (Buea and Yaoundé). In addition, DDT resistance was suspected in the *Ae. albopictus *sample from Bertoua. DDT resistance has been widely found in *Ae. aegypti *worldwide, including in Cameroon [23]. *Kdr *mutation of the voltage-gated sodium channel has been found to confer resistance to DDT in *Ae. aegypti *[39] but detoxifying enzymes such as glutathione S-transferases (GSTs) can also play a key role in DDT metabolism and resistance [40]. Although DDT resistance has also been recorded in *Ae. albopictus *in Thailand and Japan [41,42], the underlying mechanisms remain unclear.

This study also showed full susceptibility of *Ae. aegypti *and *Ae. albopictus *to deltamethrin, except in Yaoundé where the *Ae. albopictus *population showed mortality rates of around 80%, strongly suggesting resistance. Pyrethroid resistance in *Ae. aegypti *is widespread [43,15] and is usually associated with an altered amino acid sequence of the target protein, the sodium channel that confers knockdown resistance when altered (the so-called *Kdr *mutation) [39,44]. Pyrethroid resistance has also been linked to an increase in metabolic process through over-transcription of detoxification genes [21]. Although the number of studies reporting insecticide resistance in *Ae. albopictus *has increased this decade [17,41,42,45,46], few have shown evidence of pyrethroid resistance [14]. The *Kdr *mutation has been found in *Ae. aegypti *[39,16] but has never been reported in *Ae. albopictus*. Further molecular and biochemical studies are needed to identify the genetic basis of pyrethroid resistance in *Ae. albopictus *in Yaoundé. The absence of cross-resistance between DDT and deltamethrin in all mosquito populations (except *Ae. albopictus *collected in Yaoundé) suggests the involvement of metabolic resistance rather than a target site modification (*Kdr *mutation) in the sodium channel. It is unclear whether cross-resistance to deltamethrin and DDT in the *Ae. albopictus *population in Yaoundé is due to common or different resistance mechanisms.

The source of DDT and deltamethrin resistance observed here in DENV and CHIKV vectors is unclear. Indeed, in Central Africa, insecticide treatment specifically targeting *Ae. aegypti *or *Ae. albopictus *is extremely limited. It is possible that insecticides used to control other insects of medical or agricultural interest exert indirect selection pressure on these two mosquito species. For example, indoor residual spraying of DDT for malaria control [47] was suspected of favouring the selection of DDT resistance in *Anopheles *[48] as well as in *Aedes *[42,45]. Contamination of larval breeding places by insecticides used in agriculture (cotton, vegetables) has also been shown to select for DDT and pyrethroid resistance in malaria vectors [48-52]. Concerning *Ae. albopictus*, which recently spread to Cameroon [8], one cannot exclude the possibility that the invading population possessed a resistance background.

## Conclusion

This is the first study of the susceptibility status of *Ae. aegypti *and *Ae. albopictus *to various insecticides in Central Africa. The increase in dengue and chikungunya outbreaks in this region calls for more robust surveillance and vector control. Our observations could help to guide insecticide-based strategies, although broader monitoring of insecticide resistance in *Aedes *mosquitoes is needed. Further molecular and biochemical studies are also needed to determine the mechanisms involved in insecticide resistance among arboviruses vectors in Central Africa.

## Competing interests

The authors declare that they have no competing interests.

## Authors' contributions

BK and CP designed the study and monitored its implementation. BK, EN and CP participated to field sampling. BK, EN, PN carried out bioassay tests, under guidance of JE. BK, SM and CP analyzed the data. BK, SM, VC and CP wrote the manuscript which was critically revised by FC. All authors read and approved the final manuscript.
